# 2-Chloro-*N*-(3-meth­oxy­benzo­yl)benzene­sulfonamide

**DOI:** 10.1107/S1600536813018291

**Published:** 2013-07-06

**Authors:** P. A. Suchetan, B. S. Palakshamurthy, G. R. Mamatha, Vijith Kumar, N. R. Mohan, S. Sreenivasa

**Affiliations:** a Department of Studies and Research in Chemistry, U.C.S., Tumkur University, Tumkur, Karnataka 572 103, India; b Department of Studies and Research in Physics, U.C.S., Tumkur University, Tumkur, Karnataka 572 103, India; c University College of Science, Tumkur University, Tumkur, India; d Soild State and Structural Chemistry Unit, Indian Institute of Science, Bangalore, India; e Department of Studies and Research in Chemistry, Tumkur University, Tumkur, Karnataka 572 103, India

## Abstract

In the title compound, C_14_H_12_ClNO_4_S, the dihedral angle between the chloro- and meth­oxy-substituted benzene rings is 87.40 (1)°. In the crystal, adjacent mol­ecules form inversion-related dimers through strong N—H⋯O hydrogen bonds, generating *R*_2_^2^(8) loops. The dimers are further connected through two C—H⋯O inter­actions that form *C*(11) chains and *R*_2_^2^(14) loops. Aromatic π–π stacking inter­actions [centroid–centroid separation = 3.8574 (1) Å] are also observed.

## Related literature

For a similar structure, see: Gowda *et al.* (2010 ▶)
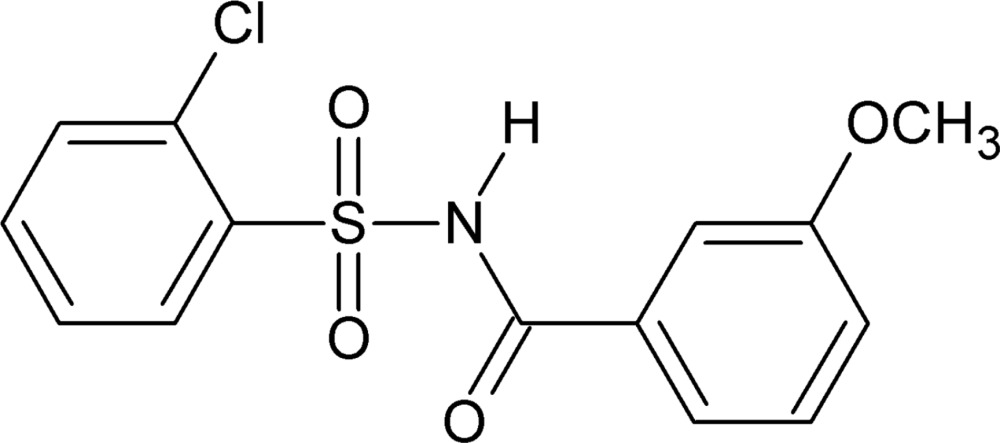


## Experimental

### 

#### Crystal data

C_14_H_12_ClNO_4_S
*M* *_r_* = 325.76
Triclinic, 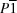

*a* = 7.5731 (5) Å
*b* = 10.1861 (5) Å
*c* = 10.3636 (6) Å
α = 94.945 (4)°
β = 96.581 (5)°
γ = 110.974 (5)°
*V* = 734.56 (7) Å^3^
*Z* = 2
Mo *K*α radiation
μ = 0.42 mm^−1^
*T* = 293 K
0.35 × 0.28 × 0.22 mm

#### Data collection

Bruker APEXII diffractometer
11351 measured reflections
2584 independent reflections
2133 reflections with *I* > 2σ(*I*)
*R*_int_ = 0.038
2 standard reflections every 1 reflections intensity decay: 10%

#### Refinement

*R*[*F* ^2^ > 2σ(*F* ^2^)] = 0.037
*wR*(*F* ^2^) = 0.101
*S* = 1.05
2584 reflections
195 parameters
H atoms treated by a mixture of independent and constrained refinement
Δρ_max_ = 0.19 e Å^−3^
Δρ_min_ = −0.30 e Å^−3^

### 

Data collection: *APEX2* (Bruker, 2009 ▶); cell refinement: *APEX2* and *SAINT-Plus* (Bruker, 2009 ▶); data reduction: *SAINT-Plus* and *XPREP* (Bruker, 2009 ▶); program(s) used to solve structure: *SHELXS97* (Sheldrick, 2008 ▶); program(s) used to refine structure: *SHELXL97* (Sheldrick, 2008 ▶); molecular graphics: *Mercury* (Macrae *et al.*, 2008 ▶); software used to prepare material for publication: *SHELXL97*.

## Supplementary Material

Crystal structure: contains datablock(s) I, global. DOI: 10.1107/S1600536813018291/hg5328sup1.cif

Structure factors: contains datablock(s) I. DOI: 10.1107/S1600536813018291/hg5328Isup2.hkl

Supplementary material file. DOI: 10.1107/S1600536813018291/hg5328Isup3.cml

Additional supplementary materials:  crystallographic information; 3D view; checkCIF report

## Figures and Tables

**Table 1:** Hydrogen-bond geometry (Å, °)

*D*—H⋯*A*	*D*—H	H⋯*A*	*D*⋯*A*	*D*—H⋯*A*
N1—H*N*1⋯O1^i^	0.78 (3)	2.14 (3)	2.926 (3)	170 (3)
C5—H5⋯O3^ii^	0.93	2.53	3.417 (3)	160
C3—H3⋯O3^iii^	0.93	2.60	3.338 (3)	137

Symmetry codes: (i) 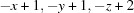; (ii) 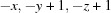; (iii) 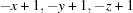.

**Fig. 1.**
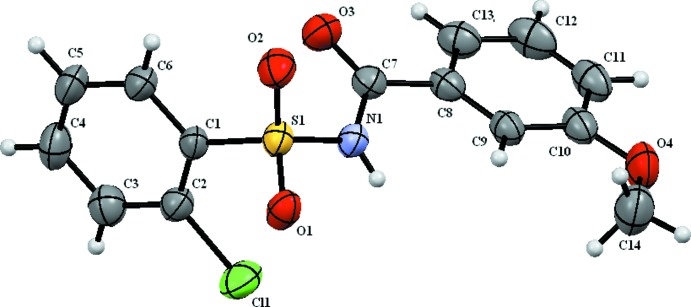
Molecular structure of the title compound, showing the atom-labeling scheme. Displacement ellipsoids are drawn at the 50% probability level.

**Fig. 2.**
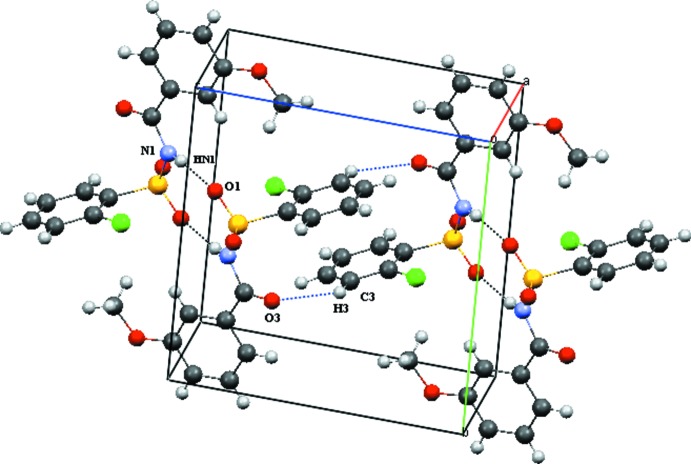
Molecular packing of (I) forming R22(8) loops and C(11) chains.

**Fig. 3.**
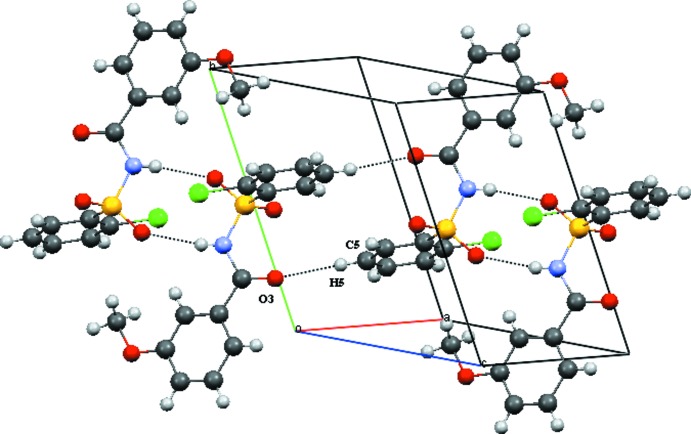
Molecular packing of (I) forming R22(14) loops.

**Fig. 4.**
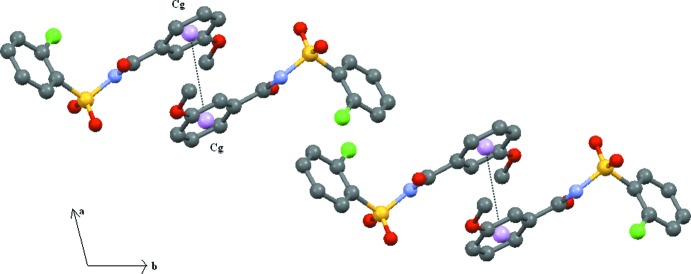
Stacking of molecules through Cg···Cg interactions. Cg is the centroid of the methoxy substituted benzene ring.

## supplementary crystallographic information

### Comment

As a part of the efforts to study the crystal structures of *N*-(aroyl)-arylsulfonamides (Gowda *et al.*, 2010), the crystal structure of the title compound (I) was determined.

In the molecule, the conformation between the N—H bond and the *ortho*-chloro group in the sulfonyl bound benzene ring is *syn*. This is similar to that observed in *N*-(benzoyl)-2-chlorobenzenesulfonamide (II, Gowda *et al.* 2010).

In the structure, the adjacent molecules form inversion related dimers through strong N—H···O hydrogen bonds, generating *R*_2_^2^(8) loops. The dimers are further connected through intermolecular C3—H3···O3 and C5—H5···O3 interactions that form C(11) chains and *R*_2_^2^(14) loops. Aromatic π-π stacking interactions (centroid-centroid separation = 3.8574 (1) Å) are also observed in the structure.

### Experimental

The title compound was prepared by refluxing a mixture of 3-methoxybenzoic acid, 2-chlorobenzenesulfonamide and phosphorous oxychloride (POCl_3_) for 2 h on a water bath. The resultant mixture was cooled and poured into ice cold water. The solid obtained was filtered and washed thoroughly with water and then dissolved in sodium bicarbonate solution. The compound was later reprecipitated by acidifying the filtered solution with dilute HCl. The filtered and dried solid was recrystallized to the constant melting point (443 K).

Colorless prisms of (I) were obtained from a slow evaporation of its ethanolic solution at room temperature.

### Refinement

The H atom of the NH group was located in a difference map and later refined freely. The other H atoms were positioned with idealized geometry using a riding model with C—H = 0.93 Å. All H atoms were refined with isotropic displacement parameters (set to 1.2 or 1.5 times of the *U*_eq_ of the parent atom).

### Crystal data

**Table:**

C_14_H_12_ClNO_4_S	*F*(000) = 336
*M**_r_* = 325.76	Prism
Triclinic, *P*1	*D*_x_ = 1.473 Mg m^−^^3^
Hall symbol: -P 1	Melting point: 443 K
*a* = 7.5731 (5) Å	Mo *K*α radiation, λ = 0.71073 Å
*b* = 10.1861 (5) Å	Cell parameters from 1029 reflections
*c* = 10.3636 (6) Å	θ = 2.7–25.0°
α = 94.945 (4)°	µ = 0.42 mm^−^^1^
β = 96.581 (5)°	*T* = 293 K
γ = 110.974 (5)°	Prism, colourless
*V* = 734.56 (7) Å^3^	0.35 × 0.28 × 0.22 mm
*Z* = 2	

### Data collection

**Table:**

Bruker APEXII diffractometer	*R*_int_ = 0.038
Radiation source: fine-focus sealed tube	θ_max_ = 25.0°, θ_min_ = 2.7°
Graphite monochromator	*h* = −8→8
phi and ω scans	*k* = −12→12
11351 measured reflections	*l* = −12→12
2584 independent reflections	2 standard reflections every 1 reflections
2133 reflections with *I* > 2σ(*I*)	intensity decay: 10%

### Refinement

**Table:**

Refinement on *F*^2^	Primary atom site location: structure-invariant direct methods
Least-squares matrix: full	Secondary atom site location: difference Fourier map
*R*[*F*^2^ > 2σ(*F*^2^)] = 0.037	Hydrogen site location: inferred from neighbouring sites
*wR*(*F*^2^) = 0.101	H atoms treated by a mixture of independent and constrained refinement
*S* = 1.05	*w* = 1/[σ^2^(*F*_o_^2^) + (0.0499*P*)^2^ + 0.1773*P*] where *P* = (*F*_o_^2^ + 2*F*_c_^2^)/3
2584 reflections	(Δ/σ)_max_ = 0.006
195 parameters	Δρ_max_ = 0.19 e Å^−^^3^
0 restraints	Δρ_min_ = −0.30 e Å^−^^3^

### Special details

**Table:**

Geometry. All s.u.'s (except the s.u. in the dihedral angle between two l.s. planes) are estimated using the full covariance matrix. The cell s.u.'s are taken into account individually in the estimation of s.u.'s in distances, angles and torsion angles; correlations between s.u.'s in cell parameters are only used when they are defined by crystal symmetry. An approximate (isotropic) treatment of cell s.u.'s is used for estimating s.u.'s involving l.s. planes.
Refinement. Refinement of *F*^2^ against ALL reflections. The weighted *R*-factor *wR* and goodness of fit *S* are based on *F*^2^, conventional *R*-factors *R* are based on *F*, with *F* set to zero for negative *F*^2^. The threshold expression of *F*^2^ > 2σ(*F*^2^) is used only for calculating *R*-factors(gt) *etc*. and is not relevant to the choice of reflections for refinement. *R*-factors based on *F*^2^ are statistically about twice as large as those based on *F*, and *R*- factors based on ALL data will be even larger.

### Fractional atomic coordinates and isotropic or equivalent isotropic displacement parameters (Å^2^)

**Table:**

	*x*	*y*	*z*	*U*_iso_*/*U*_eq_	
HN1	0.513 (4)	0.639 (3)	0.931 (3)	0.057 (8)*	
C1	0.2590 (3)	0.4163 (2)	0.67778 (18)	0.0355 (5)	
C2	0.4118 (3)	0.3814 (2)	0.6470 (2)	0.0404 (5)	
C3	0.4049 (4)	0.3179 (3)	0.5217 (2)	0.0514 (6)	
H3	0.5072	0.2947	0.5005	0.062*	
C4	0.2458 (4)	0.2894 (3)	0.4285 (2)	0.0566 (7)	
H4	0.2411	0.2460	0.3448	0.068*	
C5	0.0939 (4)	0.3242 (3)	0.4578 (2)	0.0547 (6)	
H5	−0.0122	0.3051	0.3940	0.066*	
C6	0.0999 (3)	0.3876 (2)	0.5822 (2)	0.0450 (5)	
H6	−0.0025	0.4111	0.6024	0.054*	
C7	0.4531 (3)	0.7563 (2)	0.8139 (2)	0.0385 (5)	
C8	0.6044 (3)	0.8929 (2)	0.8800 (2)	0.0390 (5)	
C9	0.6794 (3)	0.9116 (2)	1.0120 (2)	0.0402 (5)	
H9	0.6398	0.8371	1.0612	0.048*	
C10	0.8142 (3)	1.0429 (2)	1.0698 (2)	0.0450 (5)	
C11	0.8727 (3)	1.1534 (2)	0.9965 (3)	0.0533 (6)	
H11	0.9626	1.2412	1.0355	0.064*	
C12	0.7984 (4)	1.1338 (3)	0.8665 (3)	0.0585 (7)	
H12	0.8384	1.2087	0.8177	0.070*	
C13	0.6639 (3)	1.0035 (2)	0.8062 (2)	0.0506 (6)	
H13	0.6146	0.9907	0.7176	0.061*	
C14	0.8344 (4)	0.9647 (3)	1.2797 (3)	0.0710 (8)	
H14A	0.6997	0.9395	1.2805	0.106*	
H14B	0.9025	0.9995	1.3673	0.106*	
H14C	0.8580	0.8825	1.2465	0.106*	
O1	0.2842 (2)	0.39742 (15)	0.92753 (14)	0.0450 (4)	
O2	0.0743 (2)	0.51231 (17)	0.83161 (15)	0.0508 (4)	
O3	0.3527 (2)	0.74552 (17)	0.70992 (15)	0.0526 (4)	
O4	0.8980 (3)	1.07160 (17)	1.19821 (17)	0.0628 (5)	
S1	0.24743 (7)	0.48701 (5)	0.83723 (5)	0.03761 (17)	
N1	0.4298 (3)	0.63820 (18)	0.87817 (18)	0.0398 (4)	
Cl1	0.61560 (9)	0.41609 (7)	0.75991 (6)	0.0578 (2)	

### Atomic displacement parameters (Å^2^)

**Table:**

	*U*^11^	*U*^22^	*U*^33^	*U*^12^	*U*^13^	*U*^23^
C1	0.0390 (12)	0.0294 (10)	0.0326 (10)	0.0097 (9)	−0.0032 (9)	0.0000 (8)
C2	0.0387 (13)	0.0383 (12)	0.0398 (11)	0.0116 (10)	−0.0013 (9)	0.0044 (9)
C3	0.0547 (15)	0.0508 (14)	0.0481 (13)	0.0199 (12)	0.0083 (11)	0.0019 (11)
C4	0.0656 (18)	0.0565 (15)	0.0368 (12)	0.0152 (13)	−0.0003 (11)	−0.0072 (11)
C5	0.0583 (16)	0.0535 (15)	0.0409 (13)	0.0166 (13)	−0.0149 (11)	−0.0053 (11)
C6	0.0412 (13)	0.0404 (12)	0.0469 (13)	0.0134 (10)	−0.0088 (10)	0.0000 (10)
C7	0.0385 (12)	0.0381 (12)	0.0400 (11)	0.0164 (10)	0.0054 (9)	0.0027 (9)
C8	0.0383 (12)	0.0318 (11)	0.0489 (12)	0.0145 (9)	0.0096 (10)	0.0056 (9)
C9	0.0384 (12)	0.0303 (11)	0.0499 (13)	0.0110 (9)	0.0064 (10)	0.0030 (9)
C10	0.0380 (13)	0.0344 (12)	0.0598 (14)	0.0123 (10)	0.0065 (10)	−0.0022 (10)
C11	0.0426 (14)	0.0327 (12)	0.0795 (18)	0.0084 (10)	0.0126 (12)	0.0023 (12)
C12	0.0550 (16)	0.0392 (13)	0.0849 (19)	0.0141 (12)	0.0252 (14)	0.0237 (13)
C13	0.0508 (15)	0.0462 (14)	0.0584 (14)	0.0196 (12)	0.0124 (11)	0.0142 (11)
C14	0.079 (2)	0.0590 (17)	0.0580 (16)	0.0125 (15)	−0.0047 (14)	−0.0001 (13)
O1	0.0472 (9)	0.0376 (8)	0.0398 (8)	0.0055 (7)	−0.0025 (7)	0.0085 (6)
O2	0.0379 (9)	0.0558 (10)	0.0545 (9)	0.0164 (8)	0.0025 (7)	−0.0040 (8)
O3	0.0556 (10)	0.0529 (10)	0.0475 (9)	0.0212 (8)	−0.0036 (8)	0.0079 (7)
O4	0.0652 (12)	0.0421 (10)	0.0614 (11)	0.0051 (9)	−0.0064 (9)	−0.0090 (8)
S1	0.0354 (3)	0.0351 (3)	0.0362 (3)	0.0091 (2)	−0.0017 (2)	0.0003 (2)
N1	0.0388 (11)	0.0319 (10)	0.0411 (10)	0.0091 (8)	−0.0079 (8)	0.0010 (8)
Cl1	0.0433 (4)	0.0770 (5)	0.0540 (4)	0.0283 (3)	−0.0046 (3)	0.0028 (3)

### Geometric parameters (Å, º)

**Table:**

C1—C2	1.388 (3)	C9—C10	1.390 (3)
C1—C6	1.394 (3)	C9—H9	0.9300
C1—S1	1.769 (2)	C10—O4	1.365 (3)
C2—C3	1.387 (3)	C10—C11	1.382 (3)
C2—Cl1	1.732 (2)	C11—C12	1.368 (4)
C3—C4	1.379 (3)	C11—H11	0.9300
C3—H3	0.9300	C12—C13	1.390 (3)
C4—C5	1.376 (4)	C12—H12	0.9300
C4—H4	0.9300	C13—H13	0.9300
C5—C6	1.380 (3)	C14—O4	1.417 (3)
C5—H5	0.9300	C14—H14A	0.9600
C6—H6	0.9300	C14—H14B	0.9600
C7—O3	1.218 (2)	C14—H14C	0.9600
C7—N1	1.391 (3)	O1—S1	1.4337 (14)
C7—C8	1.494 (3)	O2—S1	1.4200 (16)
C8—C13	1.386 (3)	S1—N1	1.6402 (18)
C8—C9	1.389 (3)	N1—HN1	0.79 (3)
			
C2—C1—C6	119.67 (19)	O4—C10—C9	123.9 (2)
C2—C1—S1	123.23 (15)	C11—C10—C9	120.2 (2)
C6—C1—S1	116.98 (17)	C12—C11—C10	120.0 (2)
C3—C2—C1	119.8 (2)	C12—C11—H11	120.0
C3—C2—Cl1	117.89 (18)	C10—C11—H11	120.0
C1—C2—Cl1	122.28 (16)	C11—C12—C13	120.9 (2)
C4—C3—C2	119.8 (2)	C11—C12—H12	119.5
C4—C3—H3	120.1	C13—C12—H12	119.5
C2—C3—H3	120.1	C8—C13—C12	119.1 (2)
C5—C4—C3	120.9 (2)	C8—C13—H13	120.5
C5—C4—H4	119.5	C12—C13—H13	120.5
C3—C4—H4	119.5	O4—C14—H14A	109.5
C4—C5—C6	119.7 (2)	O4—C14—H14B	109.5
C4—C5—H5	120.2	H14A—C14—H14B	109.5
C6—C5—H5	120.2	O4—C14—H14C	109.5
C5—C6—C1	120.2 (2)	H14A—C14—H14C	109.5
C5—C6—H6	119.9	H14B—C14—H14C	109.5
C1—C6—H6	119.9	C10—O4—C14	118.15 (18)
O3—C7—N1	120.3 (2)	O2—S1—O1	119.16 (10)
O3—C7—C8	123.37 (19)	O2—S1—N1	109.37 (10)
N1—C7—C8	116.29 (18)	O1—S1—N1	104.24 (9)
C13—C8—C9	120.4 (2)	O2—S1—C1	107.73 (10)
C13—C8—C7	117.8 (2)	O1—S1—C1	108.22 (9)
C9—C8—C7	121.78 (19)	N1—S1—C1	107.61 (10)
C8—C9—C10	119.4 (2)	C7—N1—S1	123.60 (15)
C8—C9—H9	120.3	C7—N1—HN1	119.7 (19)
C10—C9—H9	120.3	S1—N1—HN1	116.2 (19)
O4—C10—C11	115.9 (2)		
			
C6—C1—C2—C3	−0.1 (3)	O4—C10—C11—C12	179.1 (2)
S1—C1—C2—C3	175.85 (17)	C9—C10—C11—C12	−0.2 (4)
C6—C1—C2—Cl1	179.10 (16)	C10—C11—C12—C13	0.0 (4)
S1—C1—C2—Cl1	−4.9 (3)	C9—C8—C13—C12	−0.6 (3)
C1—C2—C3—C4	−0.3 (3)	C7—C8—C13—C12	177.2 (2)
Cl1—C2—C3—C4	−179.55 (18)	C11—C12—C13—C8	0.4 (4)
C2—C3—C4—C5	0.6 (4)	C11—C10—O4—C14	176.2 (2)
C3—C4—C5—C6	−0.5 (4)	C9—C10—O4—C14	−4.5 (3)
C4—C5—C6—C1	0.1 (4)	C2—C1—S1—O2	178.82 (17)
C2—C1—C6—C5	0.2 (3)	C6—C1—S1—O2	−5.11 (19)
S1—C1—C6—C5	−175.99 (18)	C2—C1—S1—O1	−51.1 (2)
O3—C7—C8—C13	−15.7 (3)	C6—C1—S1—O1	124.97 (16)
N1—C7—C8—C13	164.6 (2)	C2—C1—S1—N1	60.99 (19)
O3—C7—C8—C9	162.0 (2)	C6—C1—S1—N1	−122.94 (17)
N1—C7—C8—C9	−17.6 (3)	O3—C7—N1—S1	−11.6 (3)
C13—C8—C9—C10	0.4 (3)	C8—C7—N1—S1	167.99 (15)
C7—C8—C9—C10	−177.34 (19)	O2—S1—N1—C7	−52.2 (2)
C8—C9—C10—O4	−179.3 (2)	O1—S1—N1—C7	179.30 (18)
C8—C9—C10—C11	0.0 (3)	C1—S1—N1—C7	64.5 (2)

### Hydrogen-bond geometry (Å, º)

**Table:**

*D*—H···*A*	*D*—H	H···*A*	*D*···*A*	*D*—H···*A*
N1—H*N*1···O1^i^	0.78 (3)	2.14 (3)	2.926 (3)	170 (3)
C5—H5···O3^ii^	0.93	2.53	3.417 (3)	160
C3—H3···O3^iii^	0.93	2.60	3.338 (3)	137

Symmetry codes: (i) −*x*+1, −*y*+1, −*z*+2; (ii) −*x*, −*y*+1, −*z*+1; (iii) −*x*+1, −*y*+1, −*z*+1.
